# Mir‐122 upregulation and let‐7f downregulation combination: The effects on hepatic differentiation of hiPSCs on the PCL‐Gel‐HA nanofibrous scaffold

**DOI:** 10.1111/jcmm.17552

**Published:** 2022-09-13

**Authors:** Maliheh Parvanak, Zohreh Mostafavi‐Pour, Masoud Soleimani, Amir Atashi, Ehsan Arefian, Elaheh Esmaeili

**Affiliations:** ^1^ Biochemistry Department, School of Medicine Shiraz University of Medical Sciences Shiraz Iran; ^2^ Autophagy Research Center Shiraz University of Medicel Sciences Shiraz Iran; ^3^ Hematology and Cell Therapy Department, Faculty of Medical Sciences Tarbiat Modares University Tehran Iran; ^4^ Department of Tissue Engineering and Applied Cell Sciences, School of Advanced Technologies in Medicine, Student Research Committee Shahid Beheshti University of Medical Sciences Tehran Iran; ^5^ Stem cell and Tissue Engineering Research Center Shahroud University of Medical Sciences Shahroud Iran; ^6^ Department of Microbiology, School of Biology, College of Science University of Tehran Tehran Iran; ^7^ Stem Cell Technology Research Center Tehran Iran

**Keywords:** hepatic differentiation, induced pluripotent stem cells, let‐7f, miR‐122, nanofibrous scaffold

## Abstract

Cell therapy and tissue engineering as promising candidates for the liver transplantation dilemma are of special interest. Induced pluripotent stem cells (iPSCs) are one of the best sources in this field, but their differentiation methods to hepatocytes have remained challenging. We transduced human iPSCs (hiPSCs) with miR‐122 and off‐let‐7f (hiPSCs^miR‐122 + off‐let‐7f^) to evaluate how they can differentiate hiPSCs to hepatocyte‐like cells (HLCs) without any extrinsic growth factor. Additionally, we studied the effect of Poly ɛ‐caprolactone‐gelatin‐hyaluronic acid (PCL‐Gel‐HA) nanofibrous scaffold as an extracellular matrix (ECM) simulator on differentiation improvement. Definitive endoderm markers (FOXA2 and SOX17), as well as hepatic markers (AFP, Albumin, CK18, HNF4α) expression, were significantly higher in hiPSCs^miR‐122 + off‐let‐7f^ derived HLCs (hiPSCs‐HLCs) compared to the control group (miR‐scramble transduced hiPSCs: hiPSCs^scramble^). hiPSCs‐HLCs indicated hepatocyte morphological characteristics and positive immunostaining for AFP, Albumin and HNF4α. Albumin and urea secretion were significantly higher in hiPSCs‐HLCs than hiPSCs^scramble^. Comparing these markers in the PCL‐Gel‐HA group with the tissue culture plate (TCP) group revealed that PCL‐Gel‐HA could improve differentiation towards HLCs significantly. Regarding our results, these microRNAs can be used to differentiate hiPSCs to the functional hepatocytes for disease modelling, drug screening and cell‐based therapy in future studies.

## 1 INTRODUCTION

Liver failure as a big dilemma in global health with an uptrend pattern has devoted the fifth cause of death to itself.^1^ Organ transplantation is often the only option to rescue the patients with end‐stage liver diseases, but because of donor shortage, immune compatibility necessity, need for lifelong immunosuppressive drug consumption, continuously growing the number of patients waiting for a donor, and despite all of these limitations the rejection risk possibility, researchers are persuaded to find alternative strategies.^2^ Therefore, cell therapy and tissue engineering could be one of the best choices, especially if cells could be used autologously.^3^ Induced pluripotent stem cells (iPSCs) are one of the best sources in this field, and their pros and cons over the ESCs and MSCs are well discussed.^4^, ^5^ They can be differentiated into any adult cell type and potentially could be utilized autologously.^5^ Although there is a long way to claim human iPSCs‐based therapy, human iPSCs‐derived hepatocyte‐like cells (hiPSCs‐HLCs) could be a miraculous candidate for liver disease modelling, drug screening studies, and even functional and transplantable HLCs for cell therapy applications. Unfortunately, to date, hepatic differentiation studies have not yet satisfied early hopes and expectations as growth factors are not time/cost‐effective.^6^ Therefore, researchers were encouraged to use alternative methods to induce differentiation. Micro‐RNAs (miRs) act as posttranscriptional regulators of gene expression and can affect the cell fate decision, so they can be applied as cell differentiation tools.^7^ Every tissue has a specific miR signature, and when it comes to the liver, miR‐122 is the most specific and abundant one and its overexpression is important for the maturation of foetal liver progenitors and is critical in vital functions of the liver.^8^ Conversely, let‐7f has been reported as a negative regulator in hepatic differentiation by targeting HNF4a, as low level of let‐7 is important for human embryonic liver development.^9^, ^10^

On the contrary, hepatocytes are anchorage‐dependent cells, so cultivating them on structures that can mimic the extracellular matrix (ECM) can improve their growth and function.^11^ The importance of these structures is because of their effect on the cell‐matrix interaction which is determinant in cellular behaviours such as cell adhesion, proliferation and differentiation.^12^ Electrospinning is a simple and high throughput technique that creates high porosity nanofibrous structures which mimic nanoscale patterns of natural ECM. Therefore, electrospun nanofibrous scaffolds can make good biological responses in cultured cells. In addition, these scaffolds could be easily manipulated based on the requirements of each study.^11^ Poly ɛ‐caprolactone (PCL) is a bioresorbable and biocompatible polyester that can support cell growth and proliferation. It goes without saying that its effective hybridization with bioactive molecules can improve its function.^13^ Gelatin (Gel) contains RGD(Arg‐Gly‐Asp) peptides that are the most eminent recognition sites in many ECM proteins that through them integrins mediate cell–cell and cell‐ECM interactions, so Gel can improve cell anchoring to the PCL surface. Hyaluronic acid (HA) is a biocompatible and biodegradable hydrophilic compound that affects proliferation, survival, movement and differentiation by inducing intracellular signal transduction. Adding HA to PCL improves cell attachment to the scaffold by increasing its polarity.^14^

This study aims to evaluate the synergistic effect of miR‐122 upregulation and let‐7f downregulation on the hepatic differentiation of hiPSCs to functional HLCs in the absence of extrinsic growth factors, and whether it could be an alternative for these time/cost‐consuming protocols in the future studies. In addition, we designed a nanofibrous scaffold by electrospinning PCL, gelatin and hyaluronic acid (PCL‐Gel‐HA) to study the hypothesis that 3D culture can improve differentiation compared to the 2D conventional tissue culture plates (TCP). Futuristically, these HLCs can be applied for future basic research, disease modelling, drug screening, cell therapy and tissue engineering studies.

## 2 MATERIALS AND METHODS

### 2.1 hiPSCs expansion

Human induced pluripotent stem cells were provided by Prof. Masoud Soleimani's research team in the Stem Cells Technology Research Center (Tehran‐Iran). hiPSCs were cultured on mitomycin‐C (Sigma‐Aldrich) inactivated mouse embryonic fibroblast (MEF) as feeder in iPSCs medium [DMEM/F12] supplemented with 20% knockout serum replacement, 0.1 mmol/L non‐essential amino acids, 2 mM L‐glutamine, 0.1 mM b‐mercaptoethanol and 10 ng/ml of recombinant human basic fibroblast growth factor (bFGF) (all from Invitrogen) at 37°C in a humidified 5% CO_2_. Then, hiPSCs colonies were detached enzymatically by 0.1% collagenase type IV (Invitrogen) and subcultured on the MEF or utilized to make the embryoid body (EB) by transferring to non‐adherent culture plate in EB medium (iPSCs medium without bFGF) for 5 days.^15^

### 2.2 Lentivirus production

hsa‐mir‐122, hsa‐let‐7f‐inhibitor(off‐let‐7f) lentiviral plasmid and negative control (scramble) vectors were purchased from Applied Biological Materials Inc (abm). Lentivirus production was performed as described previously.^16^ In brief, HEK293T cells were co‐transfected with psPAX2, pMD2.G, miR‐122 or off‐let‐7f, or miR‐scramble using calcium phosphate buffer. After 16 h media was discarded, thereafter supernatant containing viral particle was collected three times at 24 h intervals. After filtration by 0.45 μm membrane, it was concentrated using PEG‐NaCl.

### 2.3 hiPSCs transduction by hanging drop method

The hanging drop method was used for hiPSCs transduction as described before^17^ with some modifications. Briefly, hiPSCs colons were segmented into single cells and treated with concentrated viral particles with MOI = 30 in EB media to be transduced while forming EB in hanging drops for 2 days.^18^ The test group was transduced with both miR‐122 and off‐let‐7f (hiPSCs^miR‐122 + off‐let‐7f^), and the control group was transduced with miR‐scramble (hiPSCs^scramble^). Puromycin (sigma) treatment was done to remove non‐transduced cells. Treated cells were followed for 21 days and were analysed by different assays on the 1st, 7th, 14th and 21st days.

### 2.4 Preparation of the scaffold

Poly ɛ‐caprolactone was dissolved in chloroform‐DMF (8:2) by stirring overnight at room temperature, and the overall concentration of PCL in the solution was 12 w/v. Gel and HA were dissolved in acetic acid 40% in 20 w/v % and 0.5 w/v % final concentration, respectively. PCL and Gel‐HA were loaded in two separate syringes. PCL‐Gel‐HA nanofiber mats were made by a tow‐nozzle electrospinning procedure (flow rate = 0.3 ml/h, 500 rpm, voltage = 16 kV and 18 kV for PCL and Gel‐HA, respectively, at the 15 cm distance between nozzles and rotating drum collector). The electrospun nanofibers which were collected on a rotating drum collector on aluminium foil were dried in a vacuum overnight and named PCL‐Gel‐HA. Electrospun PCL‐Gel‐HA nanofibrous scaffolds were cross‐linked using glutaraldehyde bath vapours for ten‐min intervals.^19^

### 2.5 Scaffold assessment

The sessile drop method was used for scaffold hydrophilicity evaluation which is requisite for cell attachment to the scaffold. Briefly, the contact angle was measured on the tenth second at room temperature that the smaller the contact angle the more hydrophilicity would be. Scanning electron microscopy (SEM) analysis was done (Philips XL30) to check the morphology and structure of electrospun PCL‐Gel‐HA nanofibers.

Cell adhesion capability to the scaffold was assessed by 4′, 6‐diamidino‐2‐phenylindole (DAPI) staining at three and 72 h after cell seeding. Fluorescent images were recorded by a Nikon TE2000 microscope. Moreover, cell adhesion was evaluated by MTT assay at 1st, 2nd and 3rd h after cell seeding. The absorbance was measured at 570 nm using a plate reader (Tecan). To follow cell proliferation, the MTT test was done on the 1st, 3rd and 5th days after cell seeding.^20^

### 2.6 Hepatic differentiation by extrinsic growth factors

Human induced pluripotent stem cells to hepatocytes differentiation protocol was performed as previously described^21^ using B27 (Invitrogen), activin A (Peprotech) (Invitrogen), BMP4 (Peprotech), FGF2 (Invitrogen), hepatocyte growth factor (HGF) (Peprotech), Oncostatin‐M (Stemgent) and dexamethasone (Sigma‐Aldrich), and considered as growth factor hiPSCs^GF^ group. Cell morphology in culture plates and PCL‐Gel‐HA were recorded by an inverted microscope and SEM, respectively.

### 2.7 RNA extraction and quantitative real‐time PCR

To evaluate hepatic‐specific genes, total RNA was extracted from treated hiPSCs using RNeasy Mini Kit (Qiagen) based on the manufacturer's protocol. Extracted RNA was reverse transcribed to complementary DNA (cDNA) by cDNA Synthesis Kit (Intron), following the manufacturer's protocol. The qRT‐PCR was performed using a SYBRGreen kit (Takara) by the ABI Light Cycler (ABI step one). Three‐step procedure PCR was done for SRY‐related HMG‐box (Sox17), Forkhead box protein A2 (FOXA2), alpha‐fetoprotein (AFP), albumin (Alb), cytokeratin 18 (CK18), cationic amino acid transporter 1 (CAT‐1) and hepatocyte nuclear factor4a (HNF4a), (primers sequences, Table 1). Each PCR reaction was performed in triplicate, and data were analysed by the comparative CT method (DDCT).

**TABLE 1:** Primer Sequences and conditions used for qRT‐PCR

Primer	Sequence	T_m_ (°C)
18SrRNA	F 5′‐AGG AAT TCC CAG TAA GTG‐3′	62.9
	R 5′‐GCC TCA CTA AAC CAT CCA‐3′	60.1
Sox17	F 5′‐CAA GAT GCT GGG CAA GTC‐3′	60.2
	R 5′‐TGG TCC TGC ATG TGC TG‐3′	60.1
FoxA2	F 5′‐AGC GAG TTA AAG TAT GCT GG‐3′	58.3
	R 5′‐GTA GCT GCT CCA GTC GGA‐3′	58.2
AFP	F 5′‐TGC AGC CAA AGT GAA GAG GGA AGA‐3′	62.9
	R 5′‐CAT AGC GAG CAG CCC AAA GAA GAA‐3′	62.5
Albumin	F 5′‐TGC TTG AAT GTG CTG ATG ACA GGG‐3′	62.8
	R 5′‐AAG GCA AGT CAG CAG GCA TCT CAT C‐3′	63.5
CK18	F 5′‐CTT CTT TGA CCC AGA TGC CAA G‐3′	55.2
	R 5′‐GAG TCA TAC TGG CGG TCG TTG‐3′	55.6
HNF4α	F 5′‐TGG CGA GGA CTT TAA TCT TGG‐3′	58.6
	R 5′‐CTC AGA ACT TTG GTG TCA TTG G‐3′	58.9
CAT‐1	F 5′‐ CCC ACC CCC ATA GCT CC ‐3′	61.3
	R 5′‐CTC AGA ACT TTG GTG TCA TTG G‐3′	61.1

For miRNAs expression studies, total RNA was extracted by TRI reagent (Sigma) and reverse transcribed to cDNA using a universal cDNA synthesis kit (Exiqon, Woburn, Massachusetts), according to the manufacturer's protocol. miR‐122 and let‐7f were analysed relative to snord as the internal control.

### 2.8 Immunofluorescent staining

Poly ɛ‐caprolactone‐gelatin‐hyaluronic acid samples were fixed with paraformaldehyde/PBS (4% [w/v], Sigma‐Aldrich) for 30 min and then were dehydrated gradually in ethanol. TCP samples were fixed with 4% (w/v) paraformaldehyde (Sigma) in PBS for 20 min at 4°C. After washing, all samples were permeabilized with 0.1% Triton X‐100 (Sigma) and blocked with 10% goat serum (Sigma). Cells were then incubated overnight at 4°C with 1:200 monoclonal anti‐human albumin (Abcam), 1:200 monoclonal anti‐human alpha 1 fetoprotein (Abcam) and 1:100 monoclonal anti‐human hepatocyte nuclear factor 4 alpha (HNF4α) (Abcam); the next day samples were washed three times with PBS and incubated with secondary antibodies include phycoerythrin‐conjugated antibody (PE, 1:100; R&D, F0102B) and fluorescein‐conjugated antibody (FITC, 1:100; R&D, F0103B). Afterwards, DAPI was used for nuclear staining. Finally, cells were visualized by an inverted fluorescence microscope (Nikon 200).^22^

### 2.9 Glycogen staining

Poly ɛ‐caprolactone‐gelatin‐hyaluronic acid samples were fixed with paraformaldehyde/PBS (4% [w/v], Sigma‐Aldrich) for 30 min and and then were dehydrated gradually in ethanol. TCP samples were fixed with 4% (w/v) paraformaldehyde (Sigma) in PBS for 20 min at 4°C. Glycogen storage of treated cells was assessed by periodic acid‐Schiff (PAS) staining. First, oxidation was performed with 1% periodic acid for 10 min, and cells were then washed again with PBS. Subsequently, cells were treated with Schiff's reagent (Sigma) for 15 min; after rinsing in H_2_O, haematoxylin was used for counterstaining. Finally, cells were observed under an inverted microscope.^23^

### 2.10 Urea synthesis and albumin production assay

Cell supernatant from PCL‐Gel‐HA and TCP was collected on Days 1, 7, 14 and 21 during the differentiation process and stored at −20°C. A colorimetric assay kit (Pars Azmun Co.) was used to detect urea in cell culture media. The albumin content of the culture supernatant was determined using a human ALB ELISA kit (Bethyl, E88‐129) according to the manufacturer's protocol.^15^

### 2.11 Statistical analysis

All experiments were carried out at least triplicate independently, and the data are reported as mean ± standard deviation (SD). Results were analysed by *t*‐test, two‐way analysis of variance (anova) and Bonferroni's post hoc test by GraphPad Prism 6 software (GraphPad Software Inc.). In between the comparison groups, a value of *p* < 0.05 was considered statistically significant.

## 3 RESULTS

### 3.1 Scaffold Physicochemical evaluation

**FIGURE 1:**
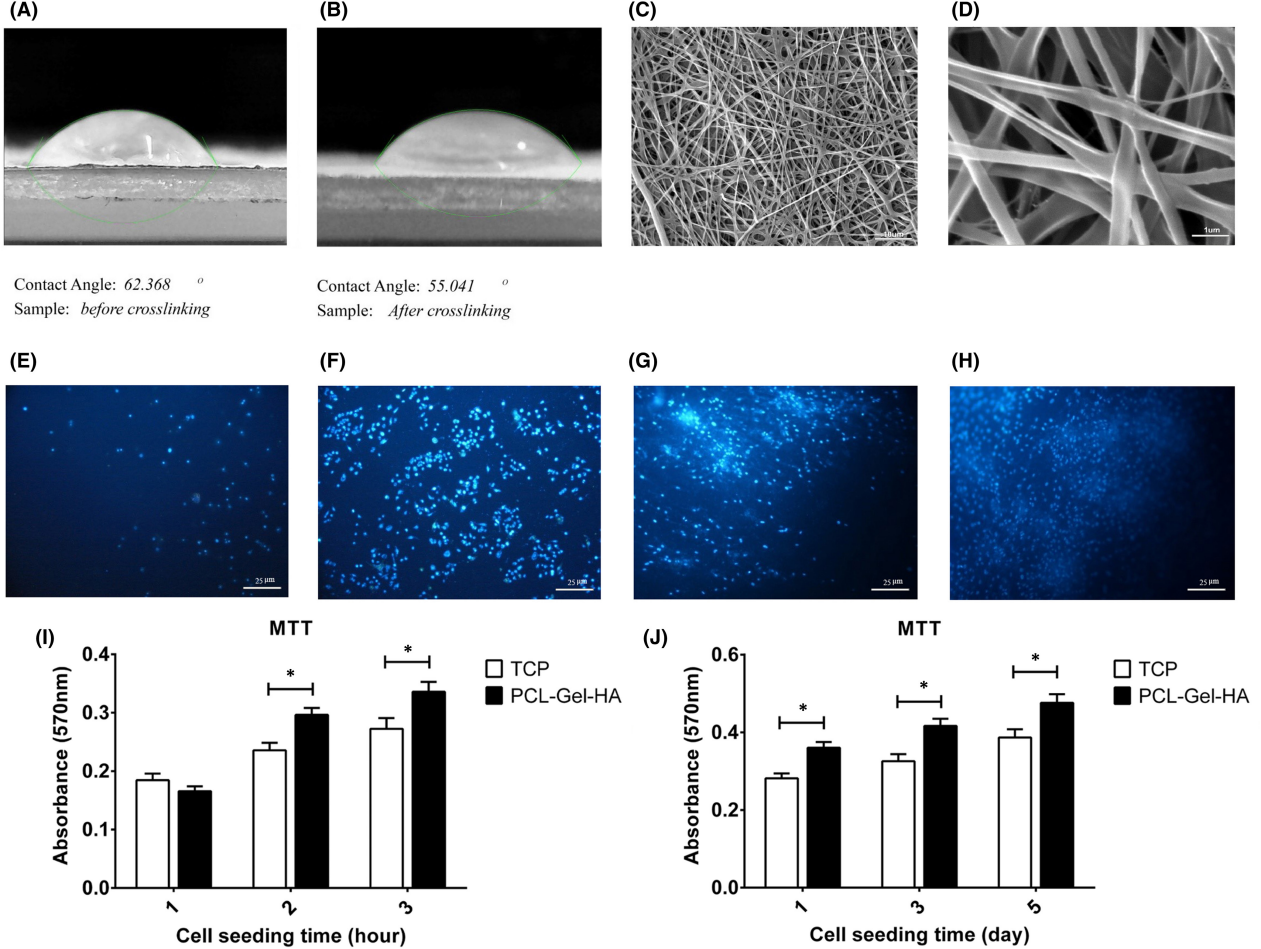
Scaffold assessment. (A and B) Sessile drop method for measuring PCL‐Gel‐HA contact angle. (C and D) Scanning electron microscopy images of nanofibrous scaffolds electrospun from PCL‐Gel‐HA after cross‐linking. DAPI staining for visualization of attached TEBs cells on (E and F) TCP after 3 and 72 h, (G and H) PCL‐Gel‐HA after 3 and 72 h. (I) MTT assay of TEBs attachment on the TCP and PCL‐Gel‐HA after 1, 2 and 3 h of cell seeding. (J) MTT assay of TEBs proliferation on the TCP and PCL‐Gel‐HA 1, 3 and 5 days after cell seeding, (*n* = 3) *p ˂ 0.05.

The sessile drop method was used to evaluate scaffold hydrophilicity. Water contact angle decreased after cross‐linking, and as cells adhere better to hydrophilic materials, so cross‐linking not only increased scaffold cohesion but cell attachment too (Figure 1A,B). SEM images of the PCL‐Gel‐HA revealed that the scaffold contains randomly oriented, uniformly sized fibres (Figure 1C,D).

### 3.2 Cell adhesion and proliferation on the PCL‐Gel‐HA

4′, 6‐diamidino‐2‐phenylindole staining images indicated that a larger number of TEB single cells (TEBCs) attached to PCL‐Gel‐HA compared to TCP both in 3, and 72 h after cell seeding(Figure 1E‐H). Figure 1I demonstrates how cell adhesion can be affected by contact surfaces. TEBC attachment to the TCP was not time‐dependent and did not increase significantly in 3 h (*p* ˃ 0.05), but TEBCs attached more rapidly to the PCL‐Gel‐HA and their attachment increased significantly over time (*p* ˂ 0.05). Besides, the MTT assay revealed that PCL‐Gel‐HA did not have toxicity for TEBCs which in turn did not have a negative effect on cell viability and also could enhance cell proliferation significantly compared to TCP (*p* ˂ 0.05, Figure 1J).

### 3.3 Transduction efficiency

**FIGURE 2:**
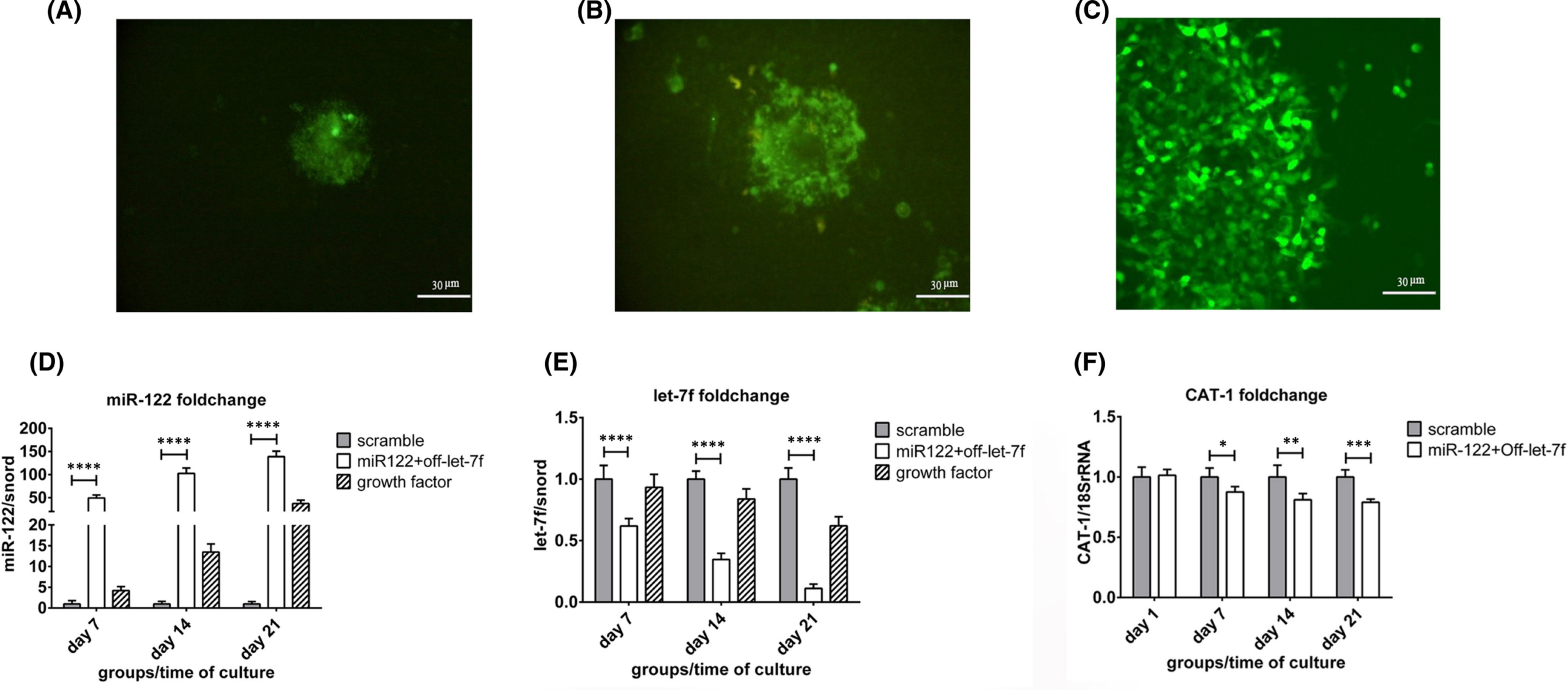
Transduction evaluation. (A–C) Lentiviral transduction of hiPSCs by miR‐122 + off‐let‐7f/GFP on Days 2, 4 and 6 after transduction. (D and E) qRT‐PCR analysing of miR‐122 and let‐7f during hepatic differentiation of hiPSCs^miR‐122 + off‐let‐7f^. Data were normalized by snord and expressed relative to scramble group. (F) qRT‐PCR results of CAT‐1 expression level in hiPSCs^miR‐122 + off‐let‐7f^ or hiPSCs^scramble^. Data were normalized with 18SrRNA and expressed relative to scramble group. The results are represented as mean ± SD (*n* = 3), *p ˂ 0.05, **p ˂ 0.001, ***p ˂ 0.001, ****p ˂ 0.0001.

As shown in Figure 2A, 48 h after transduction of the cells, a scatter pattern of GFP^+^ cells especially in the EBs margin was revealed, while GFP^+^ cells were not seen in the blank group (no transduced). On the 4th day after transduction Figure 2B, nearly half of the cells were GFP^+^, when puromycin treatment triggered and untransduced cells were removed from the population, insofar as, at the 6th day of transduction Figure 2C, almost all the remain cells were GFP^+^. The average amount of hiPSCs transduction was 48.352% by flow cytometric analysis *on* 72 h after transduction. Moreover, qRT‐PCR results were in line with fluorescent microscopy and confirmed transduction efficiency. miR‐122 expression level showed an uptrend in the hiPSCs^miR‐122 + off‐let‐7f^ group, and its expression level was significantly higher than the scramble group (*p* ˂ 0.0001); conversely, let‐7f had a descending pattern, and its expression level in the miR‐122^+off‐let‐7f^ group was significantly lower than scramble group (Figure 2D and E). To further determine whether miR‐122 has remained over‐expressed till the end of differentiation and has worked properly, we also measured CAT‐1 expression, a well‐known target of miR‐122, which is down‐regulated in response to miR‐122 overexpression (Figure 2F). CAT‐1 expression diminished in the hiPSCs^miR‐122 + off‐let‐7f^ group from the 7th day and this reduction continued, as on the 21st day it was significantly lower compared to the scramble group (*p* ˂ 0.001). In addition, we measured the miR‐122 level in the hiPSCs^GF^ group during differentiation which showed a time‐dependent manner and reached its highest level when cells showed the most amount of hepatic makers' expression on the 21st day (Figure 2D).

### 3.4 Evaluation of the morphological changes in the differentiated cells

**FIGURE 3:**
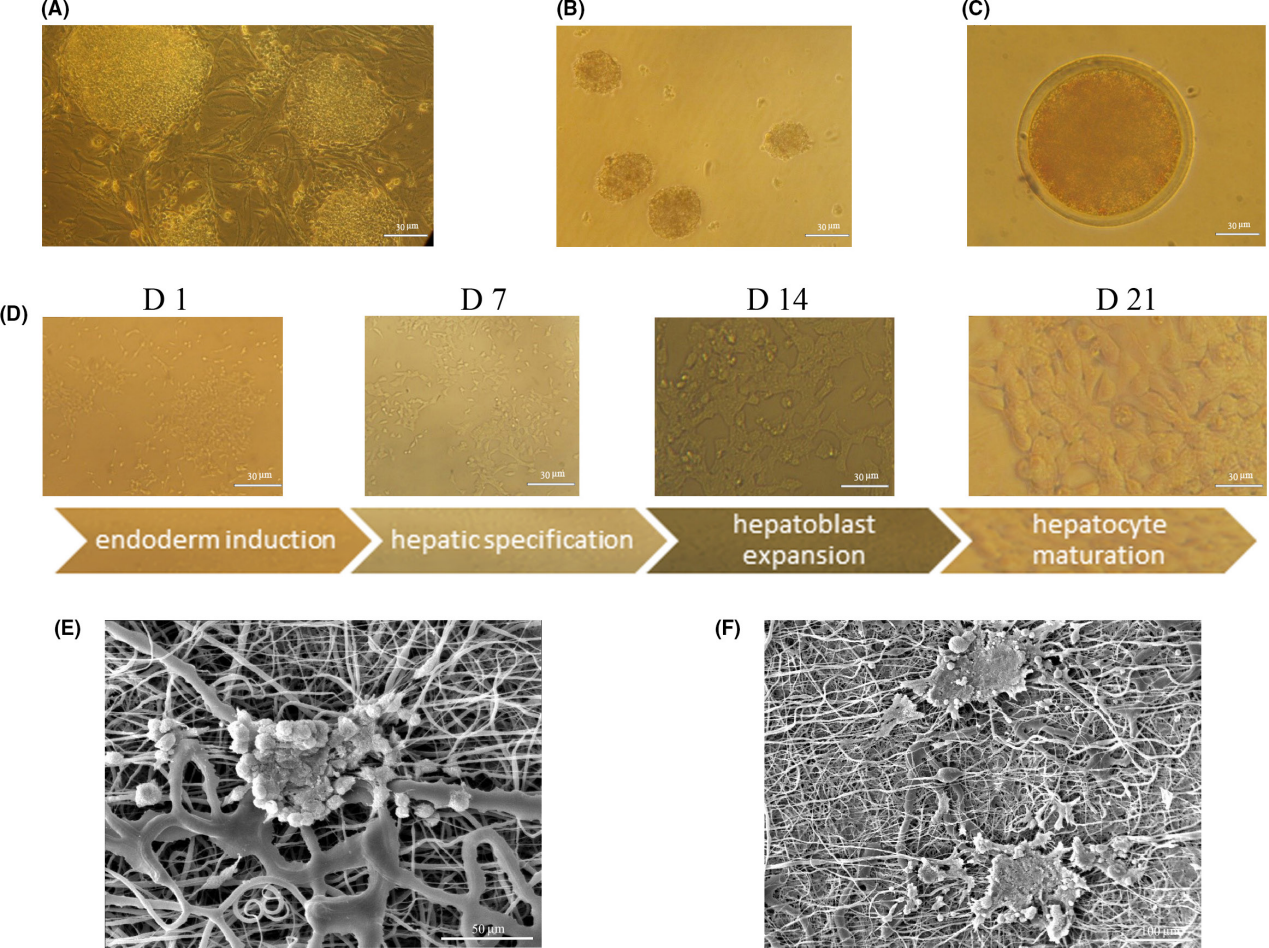
Sequential differentiation of hiPSCs^miR‐122 + off‐let‐7f^ towards HLCs. (A) hiPSCs on the MEF. (B and C) TEBs, Days 2 and 4 after transduction. (D) Morphological changes of hiPSCs^miR‐122 + off‐let‐7f^ towards HLCs on TCP. (E and F) hiPSCs on the PCL‐Gel‐HA before and after differentiation.

Six days after treatment (that was considered the first day of differentiation), morphological change started in both groups. Spheroid compacted EBs dispersed and cells gradually dissociated from each other. During the first week, cells began to change to spindle‐like cells from small round cells. Gradually cells broadened more, took a polygonal shape, and granules accumulated in their cytoplasm which increased daily till the 21st day when cells had been polygonal and cubical with a large granulated cytoplasm, a typical morphology of hepatocytes (Figure 3A–D). Morphological changes in the hiPSCs^miR122 + off‐let‐7f^ group and hiPSCs^GF^ group had almost similar progress, but these changes were not observed in the scramble group. SEM images revealed that TEBCs could penetrate and adhere well to the PCL‐Gel‐HA surface confirming the biocompatibility of these scaffolds. After 21 days, cells took a polygonal morphology like the ones on the TCP (Figure 3E and F).

### 3.5 Hepatic differentiation evaluation

To investigate how hiPSCs^miR122 + off‐let‐7f^ transduction can differentiate hiPSCs into HLCs, we analysed some endodermic and hepatic markers' mRNA levels (Sox17, FoxA2, AFP, Albumin, CK18 and HNF4α) at regular intervals during differentiation (Days 7, 14 and 21). For all the markers, there was no significant difference between the scramble group and hiPSCs which showed that lentiviral vectors and plasmid backbone did not have any effect on the differentiation process and the scramble group can be used as a negative control.

**FIGURE 4:**
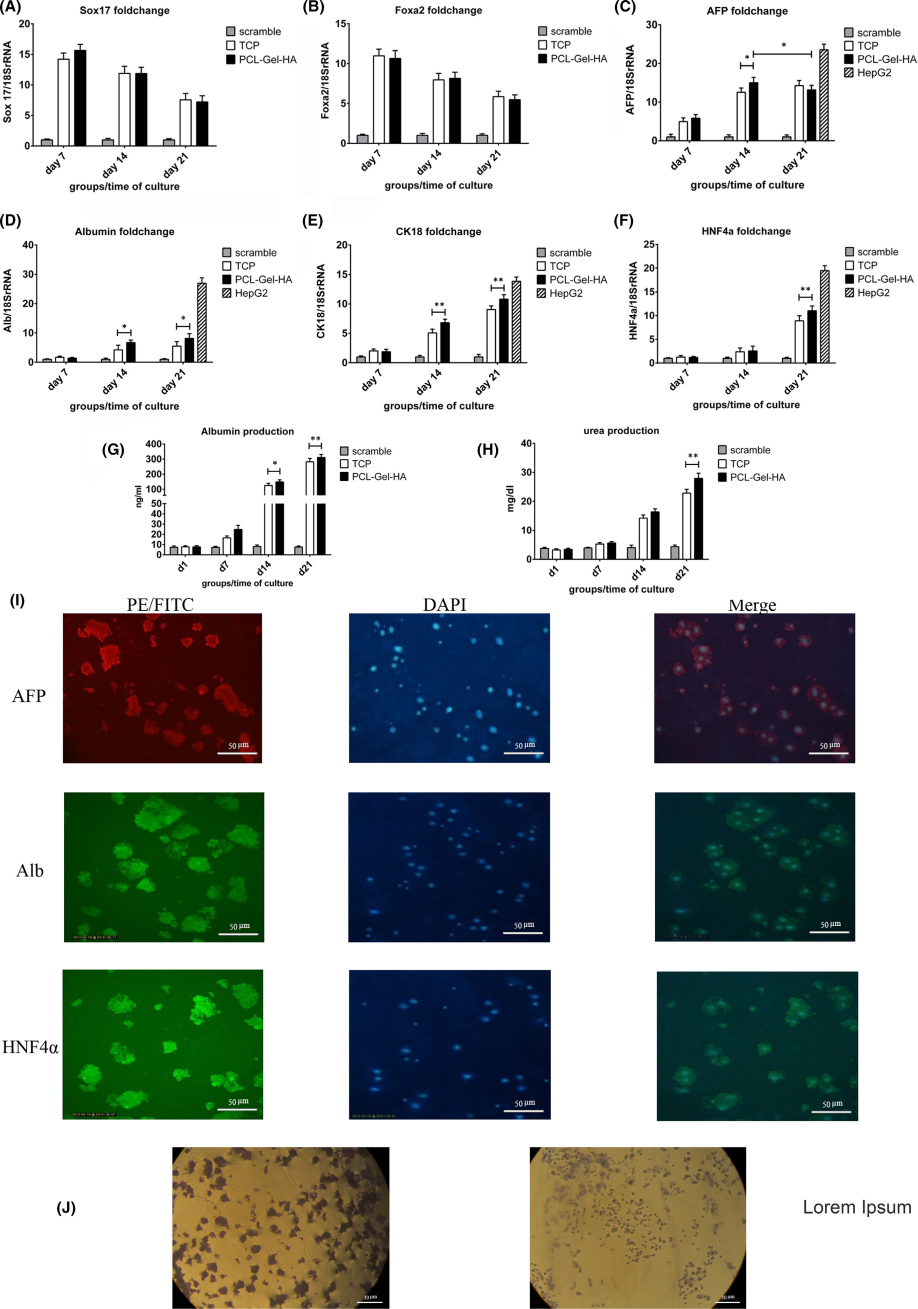
Hepatic differentiation assessment. (A–F) qRT‐PCR analysing of hepatic markers at different time points in hiPSCs^miR‐122 + off‐let‐7f^. Data were normalized by 18SrRNA and were expressed relative to the scramble group. (G and H) Albumin secretion and urea production in the hiPSCs‐HLCs supernatant compare to scramble group. (I) Immunocytochemistry assay for AFP, Albumin and HNF4α in hiPSCs‐HLCs on the 21st day of differentiation. (J) PAS staining for indicating glycogen deposition in hiPSCs‐HLCs on the 21st day of differentiation. The results are shown as mean ± SEM (*n* = 3). *p ˂ 0.05, **p ˂ 0. 01.

In the transduced cells, Sox17 and FoxA2 expression levels were significantly (*p* < 0.0001) higher than the control group during differentiation, and their maximum expression was on the 7th day of differentiation. AFP augmentation started from the first stage of differentiation (7th day) but just on the 14th and 21st days of differentiation, it was significantly (*p*˂0.0001) higher than the scramble group. In the case of albumin, we had a significant increase from the 14th day while at the first differentiation phase there was not any significant difference between transduced group and scramble. On the 14th and 21st days, transduced cells expressed significantly (*p* ˂ 0.0001) more copies of albumin transcript compared to the scramble group. The CK18 expression pattern was like albumin while HNF4α behaviour was completely different as there was notable enhancement compared to the scramble group only on the 21st day; however, HNF4α mRNA level was significantly (*p* ˂ 0.0001) higher in the transduced group compared to the scramble group, (Figure 4A–F); some of the * are not shown on the graphs.

Besides, we intended to study whether PCL‐Gel‐HA can improve hepatic differentiation of hiPSCs^miR‐122 + off‐let‐7f^. Except for the Sox17 and FoxA2 that did not show any significant difference, with the same pattern but higher amounts for other markers, PCL‐Gel‐HA caused better differentiation than did TCP. AFP mRNA level in the PCL‐Gel‐HA group on the 14th day was significantly (*p* ˂ 0.05) higher than TCP group. Albumin and CK18 expression of the PCL‐Gel‐HA group on the 14th and 21st days were significantly (*p* ˂ 0.05, *p* ˂ 0.01, respectively) higher than the TCP group. HNF4a just on the 21st day had significantly (*p* ˂ 0.01) higher expression in the PCL‐Gel‐HA group compared with the TCP group. All the results were normalized towards 18SrRNA and compared with the scramble group as the negative control. To confirm gene expression results, we investigated protein production for AFP, albumin, and HNF4α by immunocytochemistry (ICC) on the 21st day of differentiation. Positive staining for three markers was detected in HLCs differentiated from hiPSCs^miR‐122 + off‐let‐7f^ which was consistent with our gene expression results (Figure 4I).

### 3.6 Functional characteristics of hiPSCs‐derived hepatocytes

Hepatic metabolic functionality of HLCs was evaluated by measuring albumin secretion and urea production in the cells at different intervals during differentiation (on Days 1, 7, 14 and 21). The albumin level was not remarkable until the 14th day when the PCL‐Gel‐HA group produced significantly more albumin than the TCP group (*p* ˂ 0.05), and this precedence was observed on the 21st day too (*p* ˂ 0.01) (Figure 4G).

Notable urea production was observed on the 14th day without any significant difference between PCL‐Gel‐HA and TCP groups, but on the 21st day, the PCL‐Gel‐HA group had produced significantly (*p* ˂ 0.01) more urea compared to the TCP group as shown in Figure 4H.

We also performed PAS staining on the 21st day (Figure 4J) which showed glycogen deposition in HLCs cytoplasm.

## 4 DISCUSSION

In the present study, a miR‐based differentiation protocol for hepatic differentiation of hiPSCs was investigated. We demonstrated how miR‐122 overexpression alongside let‐7f downregulation can differentiate hiPSCs to HLCs without any extrinsic growth factors, as it is discussed in some studies that time/cost‐consuming growth factor‐based protocols for hepatic differentiation need to be modified or substituted with new techniques.^24^ miR could be among the best tools for differentiation induction.^25^ A group of miRs has been identified for hepatic differentiation and miR‐122 is the central member of miR‐based hepatic differentiation studies.^7^, ^26^ Cui et al.^27^ used a set of miRs for hepatic differentiation of MSCs and considered the miR‐122 group as the control group. Some studies have shown its potential in hepatic differentiation induction in foetal liver progenitor cells,^28^ ESCs^29^, ^30^ and MSCs,^31^ but as iPSCs potentially are the best source for cell‐based therapies, some advantages are raised about iPSCs.^5^, ^32^ To the best of our knowledge, two studies have investigated the role of miR‐122 in the hepatic differentiation of hiPSCs. The first one used miR‐122 and growth factors simultaneously to differentiate dental pulp iPSC into hepatocytes. The results indicated that non‐viral delivery of miR122 can shorten the time of differentiation which was started by growth factors and the delivery of miR122‐iPSC‐Heps exhibited promising hepatoprotective efficacy in vivo.^33^ Recently, another study demonstrated that miR‐122 could not differentiate hiPSCs to hepatocytes by itself and needs a contributor in this regard. They used miR‐375 overexpression as an important miR during definitive endoderm development before miR‐122 transduction and by this two‐step transduction, they could differentiate hiPSCs into hepatocytes.^16^ So we investigated miR‐122 and off‐let‐7f combination to see whether let‐7f downregulation can act as a complement for the miR‐122 hepatic differentiation role because it has been shown that let‐7f downregulation induces hepatic differentiation in MSCs, and also improve miR‐122 hepatic differentiation function in the MSCs.^34^ Moreover, previous studies have shown the inhibitory activity of the let‐7 family in hepatic differentiation^9^; Davoodian et al.^10^ have shown let‐7f reduction during hepatic differentiation of MSCs by growth factors and have discussed its negative effect on hepatic differentiation of MSCs. Our results were in line with these studies as we transduced off‐let‐7f and miR‐122 concurrently and gained functional HLCs through hepatic differentiation action of miR‐122 upregulation alongside let‐7f downregulation. As was expected according to previous reports,^16^, ^31^ we also observed CAT‐1 diminish after miR‐122 transduction and we ensured that miR transduction has worked properly, so we can impute hepatic differentiation to this miR combination.

To evaluate how 3D culture can affect differentiation we cultivated these transduced cells on the PCL‐Gel‐HA nanofibrous scaffold alongside TCP. A few studies have surveyed the effect of different 3D scaffolds on hepatic differentiation of hiPSCs, but all of them have used growth factors for differentiation induction.^15^, ^20^, ^35^ Unanimously their result revealed that simulating ECM through 3D culture can improve hepatic differentiation of hiPSCs that was evaluated by hepatic markers assessment. In this study, we have shown the same as others that hepatic differentiation can be improved in 3D conditions compared to TCP. We optimized PCL by adding Gelatin and hyaluronic acid since both of them are full of amine and carboxyl groups, are biodegradable and biocompatible, have many natural sources and are affordable.^19^, ^36^ Our results revealed that PCL‐Gel‐HA not only sufficiently supported cell attachment and proliferation, but also allows hiPSCs to differentiate to HLCs, and above all, it could improve hepatic differentiation compared to TCP. It has been discussed in hepatic differentiation reports that endoderm commitment is the first stage of hepatic differentiation.^37^, ^38^ Consistent with related reports,^16^, ^21^ we observed Sox17 and FoxA2 augmentation in the first stage of differentiation which represents endoderm induction achievement as an important step towards hepatic differentiation. Furthermore, as previous studies expressed, the expression level of hepatic‐specific markers should be investigated following hepatic differentiation induction.^15^, ^39^ We observed AFP, albumin, CK18 and HNF4α augmentation in a time‐dependent manner. All four markers revealed a higher level in the PCL‐Gel‐HA group compared to TCP which represents its improving effect on differentiation and the importance of ECM simulation in the differentiation. Of note, AFP had a different pattern. It shows decreasing pattern on the 21st day in the PCL‐Gel‐HA group (*p* ˂ 0.05), but not in the TCP group. It can be inferred that PCL‐Gel‐HA could create more mature hepatocytes compared to TCP, arguing that different studies indicated that the more mature hepatocyte, the less expression of AFP.^6^, ^21^ In another word, high expression of AFP in the late stages of hepatic differentiation is considered as a sign of immature hepatocytes. It is one of the problems with hepatic differentiation, so the methods that alleviate this immaturity could be promising.^6^ HNF4α is one of the most important hepatic transcription factors that was upregulated in our differentiated cells. At first, it was found that HNF4α binds to the miR‐122 promoter and enhances its expression.^40^ Ensuing it was clarified that it is mutual relevancy and miR‐122 can enhance HNF4α expression too.^41^ Meanwhile, let7 emerged as a hepatic differentiation suppressor through targeting HNF4α.^9^ So it can be deduced that there is a virtuous cycle between miR‐122, HNF4α and off‐let7f, and it is inferred that our proposed miR combination could be considered as a striking hepatic differentiation tool. This claim is in line with our results that an eligible hepatic differentiation was achieved without utilizing extrinsic growth factors.

Some other markers have been discussed as hallmarks of the functionality of hepatic differentiation.^21^, ^38^ As it was reported in previous studies, the results from ICC were consistent with those from qRT‐PCR.^15^, ^21^ Positive staining for AFP, albumin and HNF4α was observed that attest to gene expression results and revealed differentiation achievement. Additionally, albumin and urea production in the hiPSCs‐derived hepatocyte was another confirmation of the differentiation prosperity, and their higher level in the PCL‐Gel‐HA cells compared to TCP upholds the postulation that PCL‐Gel‐HA can intensify differentiation that has been revealed in the related studies too.^20^, ^35^ Finally, positive staining for glycogen in our derived cells was another point that previous studies considered as evidence for hepatic maturation.^23^, ^42^

To the best of our knowledge, it is the first report evaluating the hepatic differentiation of hiPSCs on the nanofibrous scaffold using miRs. Taken all together, these results support the idea that miR‐122 alongside off‐let‐7f can cause hepatic differentiation of hiPSCs without using costly extrinsic growth factors and biocompatible PCL‐Gel‐HA provides an ECM‐like microenvironment that can improve this differentiation compared to 2D conventional tissue culture plates. However, follow‐up studies are required to further determine the exact mechanism of these miRs and their synergism, and also the part played by the scaffold in the differentiation augmentation. As regard, hiPSCs potentially can be used autologously and can differentiate to all cell lineages, our proposing differentiation procedure could provide HLCs for simulating and studying liver disorders, drug screening, and with a wider vision for cell therapy and tissue engineering. Although there are some limitations for lentiviral vectors, studying new methods with more safety and efficiency to introduce these miRs to hiPSCs can be promising for clinical applications and a step, although short, towards personalized medicine.

Generally speaking, and by concluding on all the results, it can be assumed that the differentiating role of miR‐122 and off‐let‐7f besides the biomimetic function of PCL‐Gel‐HA could be responsible for the good response of hiPSCs to hepatic differentiation. This new approach could be developed and provide HLCs for cell therapy and tissue engineering.

## AUTHOR CONTRIBUTIONS

**Maliheh Parvanak:** Conceptualization (equal); data curation (equal); formal analysis (equal); investigation (equal); methodology (equal). **Zohreh Mostafavi‐Pour:** Conceptualization (equal); data curation (equal); formal analysis (equal); investigation (equal); methodology (equal); project administration (equal). **Masoud Soleimani:** Conceptualization (equal); data curation (equal); formal analysis (equal); investigation (equal); methodology (equal); project administration (equal). **Amir Atashi:** Data curation (supporting); formal analysis (supporting); methodology (supporting). **Ehsan Arefian:** Data curation (supporting); formal analysis (supporting); methodology (supporting). **Elaheh Esmaeili:** Data curation (supporting); methodology (supporting).

## CONFLICT OF INTEREST

The authors confirm that there are no conflicts of interest.

## Data Availability

The data that support the findings of this study are available from the corresponding author upon reasonable request.
